# Editorial on Special Issue “Organogels: State of the Art”

**DOI:** 10.3390/gels8030157

**Published:** 2022-03-04

**Authors:** Pablo Héctor Di Chenna

**Affiliations:** Unidad de Microanálisis y Métodos Físicos Aplicados a la Química Orgánica (UMYMFOR), Departamento de Química Orgánica, Facultad de Ciencias Exactas y Naturales, Universidad de Buenos Aires, Buenos Aires C1428EG, Argentina; dichenna@qo.fcen.uba.ar

Organogels are a very diverse and fascinating class of soft materials that, over the last 30 years, have evolved to be one of the most interesting subjects in materials science. This fact is clearly reflected by the number of references found through any web search engine, which retrieves an average of 7 references on organogels in 1990 vs. 190 references in 2021. This Special Issue overviews the current state of the art in organogels with three original papers and three reviews covering many aspects of these diverse materials from polymeric to low-molecular-weight organogelators.

All organogels have in common an organic liquid phase immobilized within a 3D entangled network built up by the gelator, and both the chemical nature of the organic liquid and the gelator drastically determine the physicochemical and mechanical properties, as well as the functionality, of these gels. Chemically speaking, organogels can be classified into two main groups: physical and chemical organogels. In chemical organogels, the organic liquid phase is trapped within an organic covalently crossed-linked polymer, and they have already found very important applications in the pharmaceutical, food, and cosmetics industries and are still under development. In this Special Issue, Kenneth P. Mineart et al. [1] report on the independence of mechanical and transport properties in organogels based on a styrenic triblock copolymer with different aliphatic mineral oils as solvents. The mineral oils used are chemically similar but have different viscosities, strongly affecting the diffusion through the organogels, a key property in transport applications such as drug delivery. In their study, they concluded that mechanical and transport properties can be independently tuned through judicious variation of the mineral oil.

On the other hand, in physical organogels—so-called supramolecular organogels—the 3D entangled network is formed by the self-assembly of small organic molecules or polymers involving non-covalent (reversible) intermolecular interactions. They have very interesting special properties compared to chemical organogels: reversibility, self-healing, and responsiveness to external stimuli, and are currently the focus of intense research. Despite the many potential industrial and high-tech applications of these materials, the organogelling process is not fully understood and is continuously under investigation. In this Special Issue, Jean-Michel Guenet [2] presents a short review on the physical properties of organogels in order to throw some light onto the gelation mechanism in terms of the thermodynamics of the process (growth mechanism and phase diagrams), morphology, molecular structure of the gelator, and rheology. The complex morphologies of the self-assembled fibrillar networks (SAFiNs) of physical organogels are often characterized by their fractal dimension; to be able to control the fractal nature of self-assembled networks is particularly important because the crystalline morphology of the organogel determines the macroscale properties such as thermostability and mechanical properties that ultimately will define the gel applications. Michael A. Rogers et al. [3] contribute to this Special Issue with a study of 1,3:2,4-Dibenzylidene sorbitol (DBS)-polyethylene glycol organogels and the influence of solvent viscosity on the SAFiN fractal growth, concluding that the solvent viscosity has a higher impact on the supramolecular fractality than the crystallization temperature, in agreement with gelation through a diffusion-limited aggregation process. The contribution of Philippe J. Mésini et al. [4] reports on the quantitative relationship between the shapes of the thermograms and phase diagrams of organogels based on 12-hydroxystearic acid in nitrobenzene, studied by micro-differential scanning calorimetry. Compared to the transition temperatures measured by other techniques, the inflection points of the thermograms measured with micro-DSC provide a melting temperature value with less bias than the temperature at the maximum of the endotherm.

The rational design of new organogelators as stimuli-responsive materials with tuned properties requires the control of the non-covalent forces directing the self-assembly, involving not only gelator–gelator but also gelator–solvent interactions. The review by Rosa M. Ortuño [5] presents a perspective on the abilities and properties of carbocycle-based organogelators, from simple cycloalkane derivatives to complex polycyclic compounds, with emphasis on the influence that chirality, conformational bias, and molecular rigidity have on their self-assembly mode and properties. Nevertheless, it is not easy, if not impossible, to predict with precision if a given compound will be able to gelate a liquid based on simple qualitative structural features. In recent years, with the development of faster computers and more accurate computational methods, several attempts have been made to simulate and predict the complex gelation process that leads to organogels. A further review by Mercedes Alonso et al. [6] provides a complete overview of research where different computational tools were used to understand the gelation process and the behavior of supramolecular gels. It can be envisaged that in the coming years, the ever-increasing computing power will enable researchers to perform calculations with higher accuracy. Thus, larger molecular events such as the self-assembly that leads to the formation of supramolecular organogels could be simulated with atomistic precision, and as a consequence, the use of computational methods will become even more prominent in the field of supramolecular organogels, helping to predict and design new gelators with tuned properties.
